# Automated design of gene circuits with optimal mushroom-bifurcation behavior

**DOI:** 10.1016/j.isci.2023.106836

**Published:** 2023-05-09

**Authors:** Irene Otero-Muras, Ruben Perez-Carrasco, Julio R. Banga, Chris P. Barnes

**Affiliations:** 1 Computational Synthetic Biology Group. Institute for Integrative Systems Biology (UV, CSIC), Spanish National Research Council, 46980 Valencia, Spain; 2 Department of Life Sciences. Imperial College London, London, UK; 3 Computational Biology Lab, MBG-CSIC, Spanish National Research Council, 36143 Pontevedra, Spain; 4 Department of Cell and Developmental Biology, University College London, London, UK

**Keywords:** Genetic engineering, Developmental biology, Biological sciences tools, Bioengineering

## Abstract

Recent advances in synthetic biology are enabling exciting technologies, including the next generation of biosensors, the rational design of cell memory, modulated synthetic cell differentiation, and generic multifunctional biocircuits. These novel applications require the design of gene circuits leading to sophisticated behaviors and functionalities. At the same time, designs need to be kept minimal to avoid compromising cell viability. Bifurcation theory addresses such challenges by associating circuit dynamical properties with molecular details of its design. Nevertheless, incorporating bifurcation analysis into automated design processes has not been accomplished yet. This work presents an optimization-based method for the automated design of synthetic gene circuits with specified bifurcation diagrams that employ minimal network topologies. Using this approach, we designed circuits exhibiting the mushroom bifurcation, distilled the most robust topologies, and explored its multifunctional behavior. We then outline potential applications in biosensors, memory devices, and synthetic cell differentiation.

## Highlights

• Optimization enables gene circuit design with target bifurcation diagrams
• The method finds robust topologies and kinetic parameters for bespoke circuit dynamics
• Optimization on the mushroom bifurcation shows cross-repression as most robust topology
• Robust mushroom topologies show capabilities as biosensor and memory devices

## Graphical abstract

**Figure:**
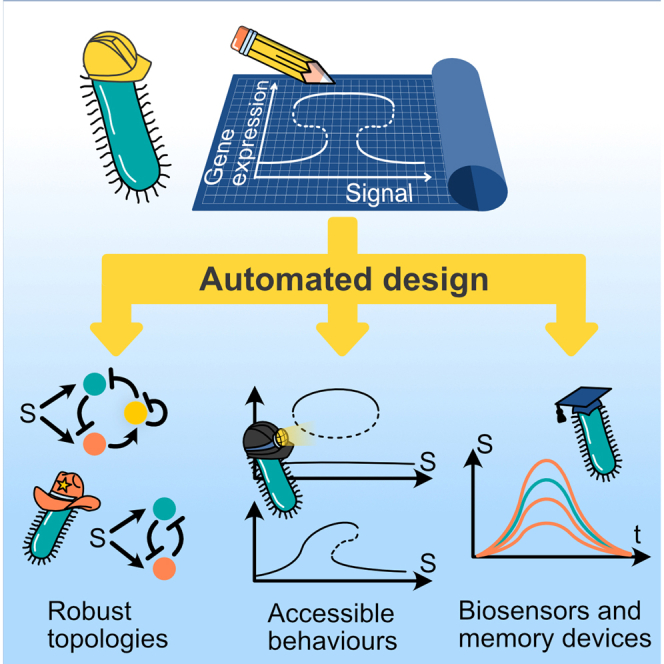

## Introduction

Real-world and cutting-edge applications of synthetic biology are demanding circuit designs with increasingly complex behaviors. Toward building the synthetic cell from the bottom-up, *new developments are expected*, quoting,^1^ *when designing cellular systems featuring complex behaviors, including division, cognition, and motility*.

One of the main challenges of synthetic biology is to design and implement gene regulatory circuits capable of complex behaviors in a near-optimal fashion while keeping a minimal design.^2^^,^^3^ A milestone in gene circuit automated design is CELLO^4^ enabling the design of circuits with pre-specified steady state and input-output behaviors, and for the first time proving good predictability in living cells of model-based automated design software. Aiming to address more complex (and dynamic) behaviors, tools based on mixed integer nonlinear programming have shown good flexibility and computational efficiency.^5^^,^^6^

The limited resources of the cell restrict the combination of multiple working circuits in the same organism.^7^^,^^8^ This gives leading relevance to the design of multifunctionality, regulatory networks capable of distinct dynamical behaviors. But, how can different behaviors be integrated in the same circuit? How to endow a cell with the capacity to respond differently to a signal leading to complex dynamical behaviors? The bifurcation theory of dynamical systems provides powerful tools to answer these questions, enabling the mapping between the topology of the network (given by a set of parametrized differential equations) and the different dynamics available under a controllable input or signal. Each bifurcation of the system changes the number and/or nature of the long-term dynamics of the system, e.g., from monostable to bistable, or from a stable steady state to an oscillator. These phenomena can be represented by bifurcation diagrams, showing how the number, position, and dynamics of each steady state change under a set of controllable parameters. However, standard tools for bifurcation analysis are based on continuation algorithms which require precise *a priori* knowledge of parameters and steady-state solutions, hampering the integration of bifurcation diagrams within automated algorithms for circuit design. Alternative methodologies based on chemical reaction network theory^9^^,^^10^^,^^11^ have paved the way for the integration of bifurcation theory into the automated design of biocircuits, an ambitious design framework that has not been addressed so far.

In this work we address this last topic and present a method for automated design of gene circuits with pre-specified bifurcation diagrams. The method extends and combines efficient global mixed integer nonlinear programming optimization methods with an innovative procedure for bifurcation detection, allowing the following novel features:

(i) Automated design of gene circuits that not only exhibit behaviors compatible with a target bifurcation diagram but are also optimized for specific additional criteria (like metabolic cost) given by sets of functions.
(ii) Systematic exploration of minimal topologies compatible with the required behavior.
(iii) Robust design i.e. finding robust topologies with respect to parameter perturbations (i.e., topologies that remain functional despite perturbations in the parameters).
(iv) More sophisticated design tasks, computing optimal trade-offs between design objectives and finding Pareto optimal topologies with respect to different opposing criteria (such as performance and metabolic cost, or robustness and topological complexity).

**Figure 1:**
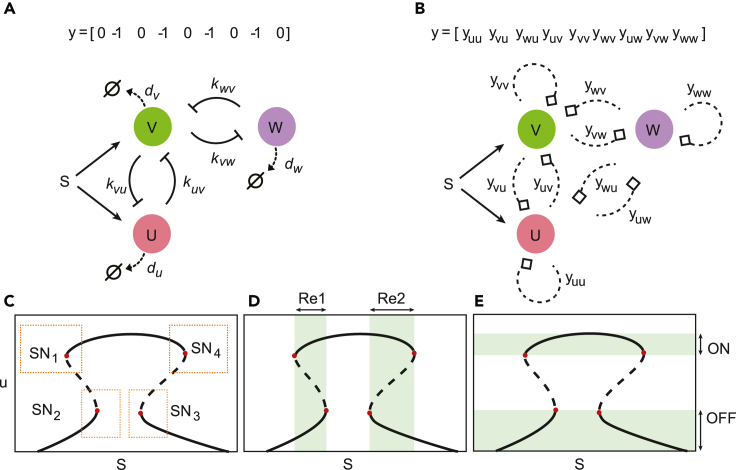
Searching for gene circuit topologies leading to mushroom bifurcation behavior (A) Gene network containing two pairs of cross-repressing genes. The affinity parameter $k_{ij}$ controls the strength of the regulatory interaction of gene *i* on gene *j*, and $d_{i}$ is the degradation rate constant for each protein species *i*. (B) The circuit superstructure employed in this work to search for mushroom behavior: super graph and vector encoding. (C) The mushroom bifurcation diagram shows four saddle-node bifurcations (indicated by red dots). The dotted squares indicate possible constraints on the state-parameter space to be imposed on the search for a desired mushroom bifurcation behavior. (D and E) (D) Two different bistability ranges (Re1, Re2) and (E) “ON state” and “OFF state” ranges (shadowed).

A multifunctional behavior of special interest for synthetic biology applications is the mushroom bifurcation, named after its mushroom-shaped bifurcation diagram, originally identified in the differentiation of neural stem cells.^12^^,^^13^^,^^14^ The mushroom can result from the combination of two toggle switches, giving rise to four saddle-node bifurcations and a set of three disconnected loci of stable steady states (Figure 1). The intermediate steady state (termed “ON state”) is only available for a window of intermediate values of the signal, while the other (termed “OFF states”) are available for high and low values of the signal. Note that despite the “ON/OFF” nomenclature, we do not require a certain threshold in the expression levels to define a mushroom. Specifically, the expression levels along each locus can vary with the signal without compromising the bifurcation diagram structure. Similarly, the pattern of steady-states of the bifurcation diagram of the mushroom is a multidimensional feature and its categorization can be independent of the gene that is used to describe it.

In certain aspects, the phenotypic behavior of the mushroom is an extension of “band-detect” gene regulatory networks, which are constructed from incoherent feedforward loops.^15^^,^^16^ These have recently been applied to model French flag type pattern formation^2^ and other programmed spatial behavior.^17^ The mushroom-shaped locus of equilibria, leading to two different ranges of bistability provides the system with unique hysteresis properties where the state of the cell will be determined by the signal history. Bistability (and multistability in general) is in itself a property of interest in synthetic biology, microorganisms, and mammalian cells,^18^^,^^19^^,^^20^ forming the basis of memory, cell decision-making systems, biological computation, and pattern formation.^21^^,^^22^^,^^23^^,^^24^ In addition, we can explore the phenotypic space close to the mushroom bifurcation. In particular, mushroom topologies can also lead to isola bifurcation diagrams, which, though well-known in dynamical systems theory, have never been demonstrated experimentally. Together, these properties demonstrate how combining common motifs gives rise to an emergent range of dynamical behaviors not described by the individual components, a growing area of interest in systems biology.^3^^,^^25^ Hence the contribution of this study is 2-fold: on the one hand incorporating for the first time target bifurcation behaviors in the automated design of biocircuits, while on the other exploring the capabilities of the identified robust mushroom topologies showing how automated exploration can be connected to functional design.

## Results

### Minimal topologies leading to mushroom bifurcation

The method presented in this work allows for the general design of biocircuits with prescribed saddle-node bifurcations in target regions of the bifurcation diagram (pre-defined by the user). Here we apply this methodology for the design of biocircuits exhibiting mushroom bifurcations with particular gene expression levels for the saddle-node bifurcations. Given that the mushroom bifurcation diagram contains four saddle-node bifurcations, we expect that a network containing two pairs of cross-repressing genes (Figure 1A) can give rise to the target bifurcation diagram. Nevertheless, we hypothesized that simpler topologies with fewer connections can also yield the same functionality. In order to explore this idea we defined the superstructure in Figure 1B. Starting from this superstructure, which includes 3 genes (U, V, and W) with a signal S inducing genes U and V, we looked for circuits with a prescribed mushroom characteristic bifurcation diagram. There are 9 potential connection arrows between the nodes (genes) represented by a vector *y* of integers, such that $y_{ij}=-1$ if gene *j* inhibits gene *i*, $y_{ij}=+1$ if gene *j* activates gene *i* and zero otherwise. The mushroom characteristic bifurcation diagram is depicted in Figure 1C, with 4 saddle-node points (SN) delimiting the steady-state branches and generating two bistability regions (Re1 and Re2 in Figure 1D). This results in an “ON state” available only for intermediate values of the signal (see Figure 1E). Note that, by “ON state” and “OFF state” we denote branches of stable the steady states in the bifurcation diagram (where “ON” and “OFF” states correspond to intermediate values of the signal, and low and high values of the signal, respectively). The particular search employed in this manuscript allows for “ON” states with lowers expression levels than the “OFF” states i.e. *inverted mushrooms*. Nevertheless, the method introduced in this manuscript allows us to restrict also the particular location of the saddle nodes (see Figure 1C), which can be especially useful in the design of specific biosensors (see STAR Methods).

We search for topologies leading to a mushroom bifurcation diagram behavior using an optimization algorithm and a multistart strategy (as described in the STAR Methods section). In order to explore minimal topologies leading to the target behavior, we first impose a number of 2 genes and a number of 2, 3, and 4 connections.

**Figure 2:**
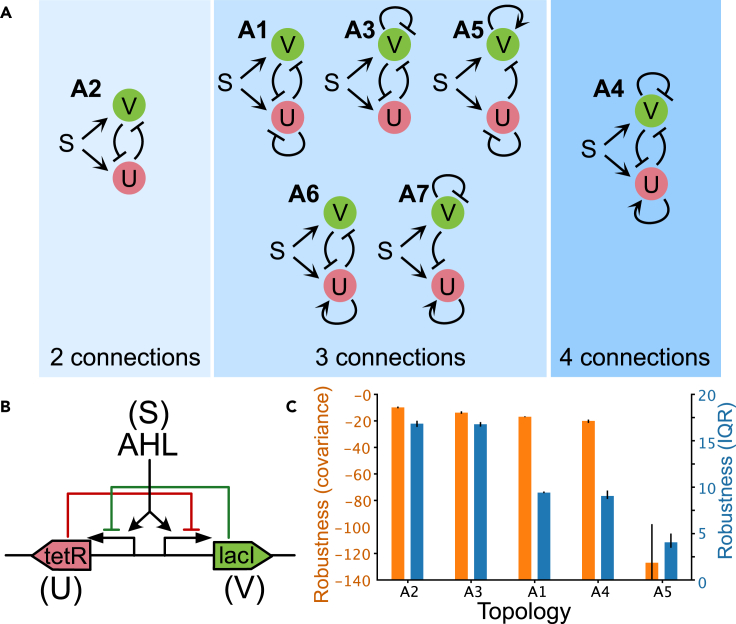
Gene regulatory circuits with 2 nodes with the capacity for mushroom bifurcation behavior (A) All the 2-gene structures found by the algorithm leading to mushroom bifurcations, labeled according to their frequency within the set of solutions (A1 and A7 are the most and least frequent, respectively). The topologies are labeled according to their frequency within the set of solutions (*Mushroom2D*). Parameter bounds for the search are included in Table S1. (B) One possible synthetic realization of the circuit with minimal topology (A2) leading to mushroom bifurcation. (C) Top 5 topologies with highest robustness (see STAR Methods). Bars and errorbars correspond with the median and quartiles from bootstrapping.

From the potential 72 connected topologies, only 7 topologies were found leading to mushroom behavior, represented in Figure 2 and classified attending to the number of active connections. Topologies, A1, A2, and A3 were the topologies that appeared more frequently in our search. This suggests that the cross-repression motif is a robust way to obtain the mushroom bifurcation diagram. In particular, the minimal structure leading to a mushroom bifurcation (A2) only requires two nodes, resembling the paradigmatic cross-repressing topology encoding the bistable switch.^18^ Note that symmetric structures such as A1 and A3 are considered different since the symmetry of the model might be broken in the particular conditions imposed. Here, we imposed a more strict range of possible values on gene *u* for the location of the saddle-node bifurcations (as indicated STAR Methods section).

In addition to the cross-repression motif, inspection of all the successful topologies revealed 2 more core topologies (for which no connection can be removed without losing the mushroom functionality), corresponding to structures A5 and A7. Interestingly, this global topology screen did not return the mushroom topology studied in^14^ and^26^ that is similar to structures A5 and A7 but without a self-activation. A specific screen targeting this topology confirmed that for the regulatory functions and parameter ranges used in our study, this topology was much less robust than the rest of the topologies found here (see Figure S4).

The key distinguishing feature for all topologies is the requirement of the signal activating both genes. A screening looking for the existence of mushroom bifurcation diagrams in the presence of a single activating input did not return any successful results, suggesting that the double activating role of the input is a requirement to reproduce the mushroom diagram.

With the same optimization strategy, we explore three-dimensional topologies leading to mushroom bifurcations by fixing the number of genes to three. The corresponding bounds for the parameters for the 3-gene network are included in Table S2. The algorithm detected through multiple optimization runs, more than 300 different 3 gene topologies leading to mushroom bifurcations from the potential 1728 topologies (the set of solutions we denote as *Mushroom3D*). Unlike exhaustive exploration strategies, our optimization-based approach finds structures fulfilling the target behavior very efficiently, in the order of seconds per run using a standard PC. The most frequent structures found are depicted in Table S3 and Figure S1. A selection of 3-gene structures which are not built up from 2-gene mushroom topologies are illustrated in Tables S4 and S5, and Figure S2.

### Robust functionality vs. topological complexity

**Figure 3:**
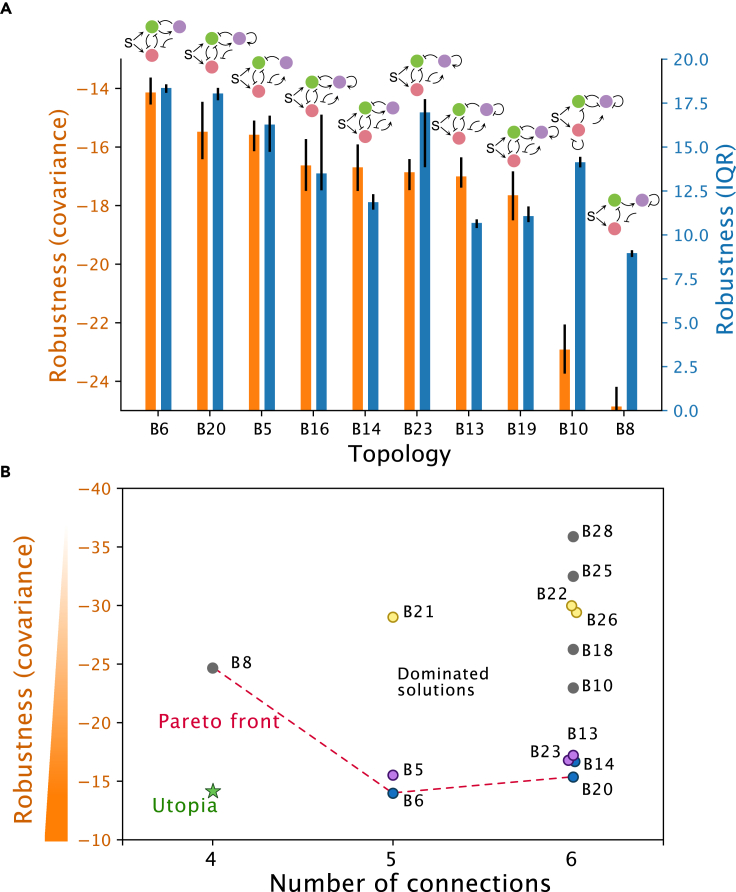
Robustness analysis for the 3-node topologies (A) Top 10 topologies with highest robustness (see STAR Methods). Bars and errorbars correspond with the median and quartiles from bootstrapping. (B) Relationship between number of connections and robustness for the most robust topologies. The Pareto front (red dashed line) is determined by the topologies with an optimal trade-off between robustness and complexity (in terms of the number of connections). Structures B8, B6, and B20 optimally trade-off robustness and complexity. Colored circles (blue, purple, and yellow) correspond with topologies that share the same 5-connection topologies (B6, B5, and B21) respectively.

A circuit is considered functional if it shows a mushroom bifurcation. Nevertheless, it is important to in additionally assess its robustness by quantifying how the circuit functionality is kept with respect to perturbations in the parameters. With this aim, we defined different robustness scores based on the size of the parameter space occupied by successful parameter sets (see STAR Methods section). The most robust structures are shown in Figures 2C and 3A. For the 2-node structures the most robust topologies show the expected cross-repression motif found in the bistable switch with marginal differences in robustness between topologies A1, A2, A3, and A4; highlighting the simplest topology A2 as a good potential candidate to construct a synthetic circuit in the lab (Figure 2B).

A similar analysis was performed for the successful topologies in 3-node networks. From the point of view of circuit implementation, we are interested in finding a good compromise between robustness and the number of connections. In order to select the best structures we build a Pareto front in the objective space (robustness vs. number of connections) as depicted in Figure 3B, identifying optimal topologies B8, B6, and B20 with 4, 5, and 6 connections, respectively. The structures in the Pareto front are shown in Figure 3B. Interestingly, the addition of connections to robust topologies did not show an improvement in the robustness of the network. In particular for the 3 most robust 5-connection topologies (B5, B6, and B21), the addition of connections just preserved or deteriorated the robustness of the network (see colored circles in Figure 3B).

### Mushroom annihilation and isola formation

**Figure 4:**
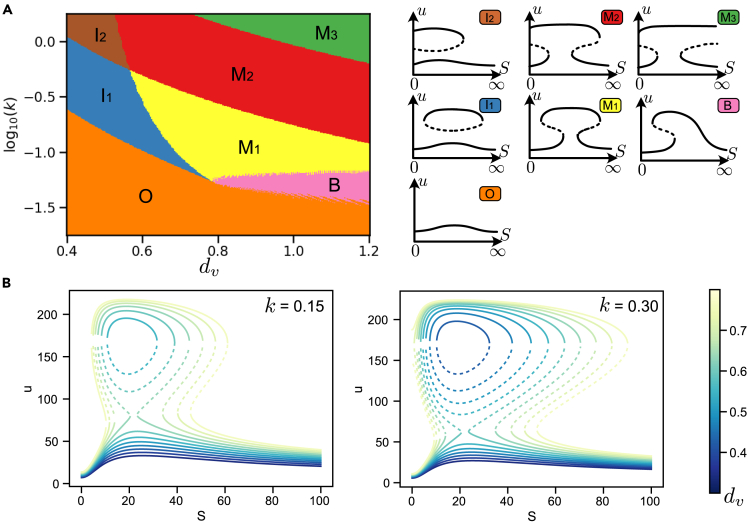
Bifurcation diagrams surrounding the mushroom behavior reveal a controllable rich functional landscape (A) Phase diagram around a mushroom generated by varying the degradation rate $d_{v}$ and the interaction strength *k* (see Figure 1A). Different colors show different bifurcation diagrams. A schematic of each bifurcation diagram is shown in the right panel indicating the different continuation combinations of stable (solid lines) and unstable states (dashed lines). (B) Change in the mushroom bifurcation diagram by changing the degradation rate $d_{v}$ for two different values of *k*. The different diagrams show the annihilation of the mushroom into an isola, followed by the collapse of the isola leading to monostability. Parameters used are: $p_{0}=230,p_{1}=50,p_{2}=1000,R_{1}=264,R_{2}=275,K_{1}=10,K_{2}=133,d_{u}=1$ for topology A3.

Following the analysis of the mushroom robustness, we explored the resulting bifurcation diagrams when the mushroom is lost. We observed three main transitions out of the mushroom bifurcation diagram ($M_{1}$ in Figure 4A): First, the mushroom head can cross values of saturation or absence or signal, resulting in incomplete mushrooms ($M_{2}$, $M_{3}$). Second, a pair of saddle nodes on one side of the mushroom collide, giving place to a cusp bifurcation giving rise to a bifurcation diagram similar to a bistable switch (*B*). Finally, the two saddle nodes forming the neck of the mushroom can collide, pinching the neck of the mushroom and producing an isola ($I_{1}$), a closed curve of equilibrium solutions delimited by two saddle nodes (Figure 4B). Strikingly, all these transitions are observed without the requirement to change the topology of the system, making this rich dynamical scenario exploration attainable in gene regulatory circuits.

While all the landscapes provide different dynamical properties, isolas are particularly compelling for their cell fate decision capabilities. Isolas are found in different contexts including chemical reactors, buckling of elastic shells and plasma physics.^27^^,^^28^ Mechanisms of birth and annihilation of isolas have been classified via singularity theory,^28^ being one of such mechanisms with particular interest in the transformation of a mushroom to an isola by reducing the number of saddle-node bifurcations.^12^^,^^29^ It is interesting that despite being found both theoretically and experimentally in chemical systems, the isola bifurcation diagram has not yet been directly observed in a biological system. Understanding how to construct such a system in a gene network could therefore enable experimental verification of the underlying dynamical theory. In addition, as we will see below, the isola can form the basis of permanent switching systems, allowing new functionalities not achievable with a genetic toggle switch.

**Figure 5:**
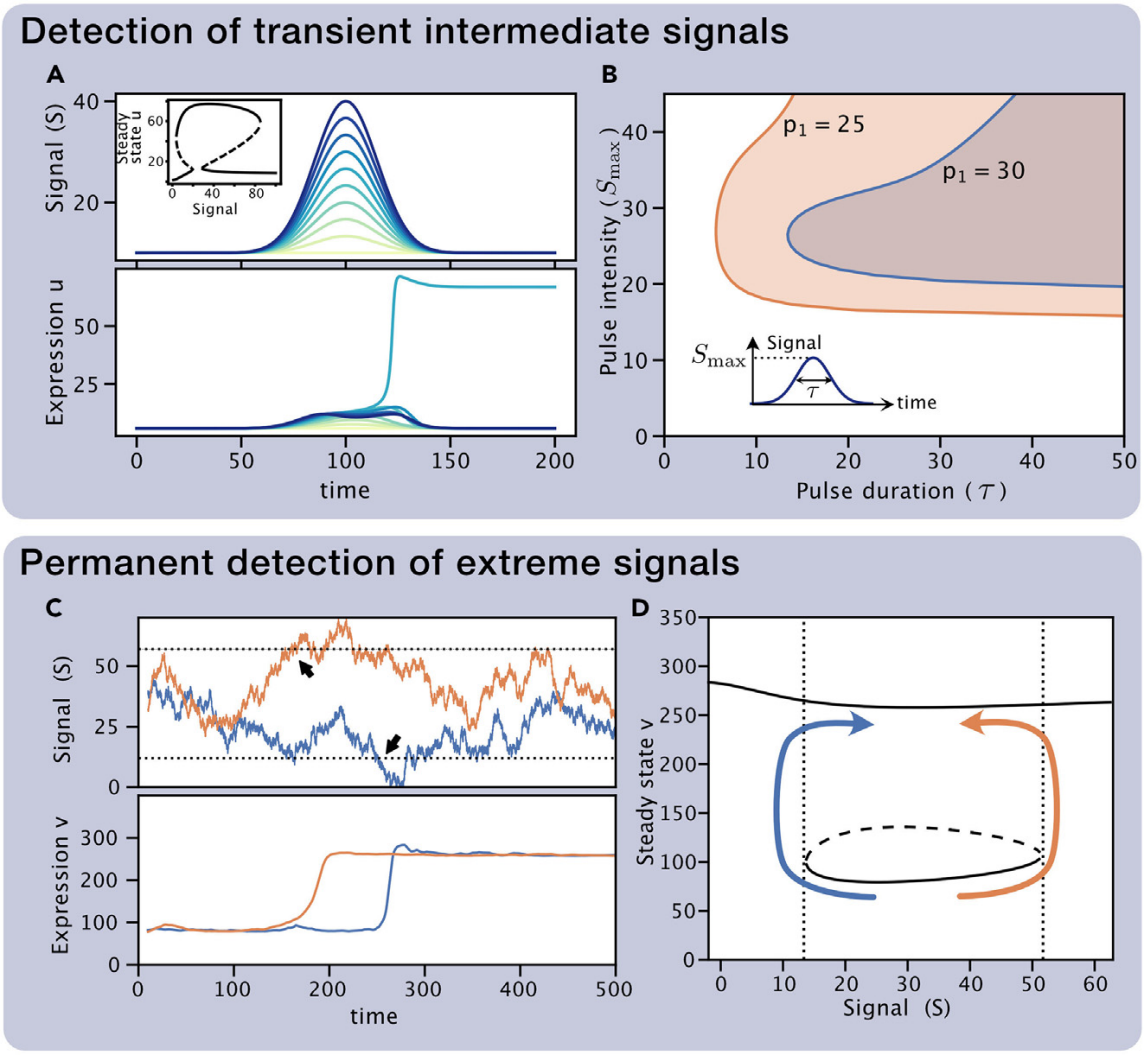
The mushroom circuit is able to discern intensities and durations of transient signals (top) It can also be used to design a sensor of extreme signal values with memory using the isola regime (bottom). (A) Response of the mushroom (inset) for different temporal signal pulses (top) of the same duration (τ=20) but different intensity (indicated by color). Expression of gene *v* (bottom) only becomes stably activated for pulses of intermediate maximum intensity ($S_{\max}$). (B) Response of the mushroom to signal pulses of different duration (τ) and maximum intensity ($S_{\max}$) for two different values of the parameter $b_{2}$. Shaded regions indicate combinations of parameters for which node *v* is activated stably. Signal profiles follow the shape $S\left(t\right)=S_{\min}+\left(S_{\max}-S_{\min}\right)e^{-\frac{1}{2}{\left(\left(t-100\right)/\tau \right)}^{2}}$ with $S_{\min}=10$. Results correspond to topology A1 with parameters $p_{0}=361,p_{1}=p_{3}=30,p_{2}=411,k=9.04\cdot 10^{-2},R_{1}=143,R_{2}=300,d_{u}=1,d_{v}=1.38,K_{1}=10.0,K_{2}=137$. (C) Expression of the circuit (bottom) for two different realizations of a noisy signal (top). (D) Isola bifurcation diagram showing the detection mechanism. When the signal reaches high or low levels determined by the isola boundary (dotted lines and black arrows in C and D), the system changes steady-state irreversibly. Schematic of the irreversible transitions is indicated by colored arrows. Results correspond to topology A1 with the same parameters than panel A but with $p_{1}=200$. Initial state was set by opening the mushroom setting parameter $p_{1}=20$. Noisy signal is a Wiener process.

Building a genetic regulatory network with a functional isola requires us to be able to prescribe the range of signals of the isola. In our parameter exploration, we observed a controllable diversity on the level and range of signals delimiting the isola. In addition, we also detected different degrees of robustness as parameters varied (Figure 5B). To explore their prevalence, we explored the formation of isolas in all the identified mushrooms’ 2D topologies and parameter sets by varying the degradation rate of the node V ($d_{v}$) and keeping the rest of the parameters constant. Strikingly, all the topologies tested exhibited the formation of isolas suggesting that the isola formation is a robust property of mushroom bifurcation. Alternatively, one can also employ the same automated method described in this paper to impose properties of the isola bifurcation diagram. In particular, these properties need to take into account, the location and curvature of the saddle-node points (see STAR Methods section for a more detailed description).

**Figure:**
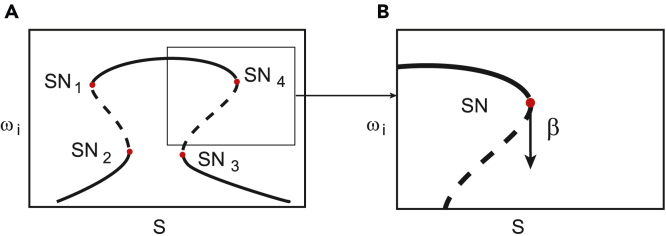
Bifurcation diagram structure used in the optimisation (A and B) (A) Mushroom-shaped bifurcation diagram and (B) Saddle-Node bifurcation and tangent vector β at the bifurcation point.

### Design of biosensors and memory devices

The bifurcation diagrams identified in the manuscript are unexplored in synthetic biology and open the door to new functionalities. One of the main characteristics of the mushroom is that the “ON state” is only available for a reduced range of signals (the neck of the mushroom). This reveals the mushroom’s capabilities as a biosensor, only allowing the activation (transition from OFF to ON) for precise levels of a target signal located in the neck of the mushroom. Furthermore, since the neck of the mushroom is surrounded by two saddle-node bifurcations, the resulting dynamical ghosts will slow down the transition to the ON state.^23^ Hence, activation is not immediate, filtering out transient signals, requiring the persistence of the target signal for a certain amount of time in order to reach the ON state (Figure 5A). In addition, the size of the head of the mushroom provides memory of the activation, preserving the ON state for a larger range of signals than the ones required for the activation (Figure 5A). As discussed in the previous section the mushroom bifurcation diagram is prone to pinching by controlling parameters such as the degradation rate of one of the genes. This provides a mechanism to regulate the size of the neck mushroom, opening the door to controlling the range of target signals as well as the required duration of such signals. This way the mushroom not only serves as an accurate signal detector but also as a timer (Figure 5B).

In addition to the mushroom bifurcation, the isola also provides compelling functionalities. In contrast to a genetic toggle switch, transitions out from the isola are irreversible. Hence, once in the isola state the system is able to detect a signal that goes outside of the range of the isola (either low or high), unable to reach back to the isola independently of future levels of the signal (Figures 5C and D). Thus the isola can serve as a sensor of extreme values with infinite memory, detecting if the signal has been high or low at any time point in the past. Since the isola bifurcation diagram is close to the mushroom diagram, reset of the system onto the isola state can be done by “opening” and “closing” the neck of the mushroom.

## Discussion

In this work, we have developed an approach to biosystem design based on the specification of the bifurcation structure (through the location of saddle nodes in the bifurcation diagram) and applied it to the case of the mushroom bifurcation of interest in developmental and synthetic biology. We found gene regulatory networks composed of two and three genes that displayed the desired behavior. In particular, we found that a system based on the genetic toggle switch incorporating a signal activating both genes was sufficient to reproduce a mushroom bifurcation diagram. We also explored the robustness of these circuits and built a Pareto front to capture the trade-off between the robustness and number of connections, identifying the best topologies to implement the circuit using synthetic biology tools. In addition, we explore specific dynamical functionalities of the mushroom that can be exploited to build biosensors with tuneable temporal and precision properties. We extended our exploration to the formation of different bifurcation diagrams, which occur in the parameter space close to the mushroom bifurcation. We showed that a variety of dynamical behaviors can be created that can provide interesting memory and signal-detecting capabilities unattainable with previous memory devices. Interestingly, this variety of behaviors was attained mainly by inducing differences in the degradation rates of the genes of the network, which is a common perturbation tool in synthetic biology,^30^ and opens the door to the exploration of novel dynamical behavior when protein degradation is coupled to other synthetic circuits.^31^^,^^32^ The most interesting bifurcation diagram found is the isola, which not only could be useful for error checking of deployed biosensors but could be useful in clinical applications, for example *in vivo* detection of inflammation^33^ or metabolite levels.^34^^,^^35^ Finally, the minimal mushroom topologies identified reveal a new way to create multistable systems, avoiding the need for loading a single bistable switch with additional autoregulation.^36^^,^^37^^,^^38^

Our results complement the small amount of other existing work on mushroom bifurcation. A recent study explored four incoherent feedforward networks with positive auto-regulation for their potential to display mushroom dynamics.^14^ They found that all were capable of mushroom-like behavior, though the appearance depended on the incoherence of the networks and the strength of positive feedback. Even though their networks apparently depend on a unique input signal, their topologies depend on an upstream gene that imposes different regulation in the downstream genes, effectively recovering the double signal input we analyze in our study. By contrast, they included the possibility of repressing input signals, and a different regulatory formalism based on AND-type logic gate interaction in the activation and repression of genes. This reinforces how the results of such computational investigations depend somewhat on the modeling choice of the dynamics, a result demonstrated directly previously.^39^ Further work in the experimental realization of these systems is required to understand the actual dynamics, and how these depend on the particular biological context. Another study modeled feedforward topologies with positive auto-regulation and demonstrated that only incoherent feedforward networks were capable of atypical bistabilities (mushrooms and isola’s).^26^ Although in this study we considered more complex topologies, we can see that when considering feedforward-like networks we also find that incoherence is required (e.g., A5 and A7 in Figure 2). Analysis of the topology studied in^26^ using the thermodynamical formalism employed in our study, revealed that the topology used by these authors can also show the mushroom bifurcation albeit in a smaller parameter region than topologies A1–A7. This manifests again the relevance that the choice of regulatory functions has on the resulting robustness of a topology.

Overall, designing biological systems based directly on bifurcation properties provides a natural tool to explore phenotype-genotype relationships, relating topologies of the network with the available dynamical behaviors. Such an approach allows for the specification of a set of functional requirements without precision of explicit integration of the differential equations. In addition, it provides a tool to design target dynamics that occur in a robust manner making these approaches key to the future of engineering of biological systems where uncertainty dominates. In future work, we will extend the method to encode different types of bifurcations (including the Hopf bifurcation) related to other complex nonlinear behaviors of interest in systems and synthetic biology.

### Limitations of the study

Future research on this topic should take into account some limitations on the use of automated bifurcation design. Firstly, the choice of regulatory functions used in the study can significantly impact the results obtained, implying that it is necessary for case-by-case precise biochemical knowledge of the specific promoter and regulatory reactions used in experimental designs of the circuit. Additionally, the study does not account for stochastic effects such as intrinsic or extrinsic noise, which may influence the bifurcation behavior of the system. Finally, other cellular processes, such as competition for protein degradation, or metabolic overload; are not considered, requiring more tailored constraints in the optimization to real-world synthetic biology scenarios.

## STAR★Methods

### Key resources table

**Table:** 

REAGENT or RESOURCE	SOURCE	IDENTIFIER
**Deposited data**		

Code used for analyses	This paper	https://doi.org/10.5281/zenodo.6024249

**Software and algorithms**		

Matlab	MathWorks	https://www.mathworks.com
Python 3	Python	https://www.python.org
Affinity Designer 2	Serif	https://affinity.serif.com

### Resource availability

#### Lead contact

Further information and requests related to code should be directed to and will be fulfilled by the lead contact, Ruben Perez-Carrasco (r.perez-carrasco@imperial.ac.uk).

#### Materials availability

This study is computational and did not generate new reagents.

### Method details

#### Gene network mixed integer modeling framework

The first step of the method proposed consists of encoding the dynamics of the gene regulatory superstructure in a mixed integer modeling framework. For models endowed with typical kinetics of the Hill or Shea-Ackers types, the state vector $\omega \in R_{\geq 0}^{N}$ contains the concentrations of the protein species involved, and the system’s dimension N is determined by the number of genes in the network. See^6^ for mixed integer modeling encodings with different kinetics. The dynamics are expressed in a mixed integer framework in terms of the vectors ω, *x*, *y* in the following form as a set of Ordinary Differential Equations:

(Equation 1) $$\frac{d\omega }{dt}=\xi \left(\omega ,x,y\right)$$

where *x*, and *y* are the vectors containing respectively the real and integer design variables. In general terms, real variables might include kinetic parameters, degradation rates, promoter strengths etc, and integer variables determine the structure and connectivity of the network.

In this study, we use the superstructure in network in Figure 1B, encoded in a mixed integer framework as follows: the gene circuit topology is characterized by a vector *y* of 9 integer variables ($y_{uu}$, $y_{vu}$, $y_{wu}$, $y_{uv}$, $y_{vv}$, $y_{wv}$, $y_{uw}$, $y_{vw}$, $y_{ww}$) such that $y_{ij}=-1$ if *j* is repressed by *i*, $y_{ji}=1$ if *i* is activated by *j*, and $y_{ij}=0$ otherwise with i,j={u,v,w}. Within this framework, a gene circuit is characterized by the vector *y* and a vector *x* containing 12 real variables coding for tunable biochemical parameters (including promoter strengths, leakiness, degradation rate constants, repression and activation affinities). We consider that the rate constants governing the strengths of the active interactions $k_{ij}$ in Figure 1B are equal (*k*). As an illustrative example, the three gene system in Figure 1A (for which $y_{uu}=y_{wu}=y_{vv}=y_{uw}=y_{ww}=0$ and $y_{vu}=y_{uv}=y_{wv}=y_{vw}=-1$) is given by:

$$\frac{du}{dt}=p_{0}\frac{p_{1}+q_{1}\left(S\right)}{1+p_{1}+q_{1}\left(S\right)+kv^{2}}-d_{u}u$$

$$\frac{dv}{dt}=p_{2}\frac{p_{3}+q_{2}\left(S\right)}{1+p_{3}+q_{2}\left(S\right)+ku^{2}+kw^{2}}-d_{v}v$$

$$\frac{dw}{dt}=p_{4}\frac{p_{5}}{1+p_{5}+kv^{2}}-d_{w}w\text{,}$$

where the state vector is denoted by ω=(u,v,w) being u,v,w the concentrations of the proteins expressed by genes U,V,W respectively; $p_{0},p_{2},p_{4}$ are the promoter strengths, $p_{1},p_{3},p_{5}$ the promoter leakiness, *k* are repression strengths and $d_{u},d_{v},d_{w}$ the degradation rate constants. The functions $q_{1}\left(S\right),q_{2}\left(S\right)$ represent the concentration of activating transcription factors regulated by an input biochemical signal *S*, given by Hill functions:

$$q_{i}\left(S\right)=R_{i}\frac{S^{2}}{K_{i}^{2}+S^{2}}\text{,}$$

where *R* is the total concentration of transcription factor, *S* is the concentration of signal inducer, *K* is the dissociation constant, and the cooperativity is 2. We assume that repressor proteins bind as dimers (or equivalently there are two operator sites). Depending on the specific design scenario, a number of assumptions can be made to reduce the dimensionality of the parameter search space, here we consider $p_{3}=p_{1}$ and $d_{u}=1$. In this way, the vector of real design variables reads $x=\left(p_{0},p_{1},p_{2},p_{4},p_{5},k,R_{1},R_{2},d_{v},d_{w},K_{1},K_{2}\right)$, and the dynamics is expressed in terms of the vectors ω, *x*, *y* in the form given by Equation 1.

Importantly, previous to the formulation of the design as an optimization problem, we have to select an appropriate bifurcation parameter. We use the concentration of the inducer input *S* as bifurcation parameter and, for convenience, we express the dynamics in the compact form:

(Equation 2) $$\frac{d\omega }{dt}=f\left(\omega ,x,y,S\right)\text{.}$$

#### Bifurcation conditions

Once the dynamics are encoded in the mixed integer form (2), we formulate the conditions for the target bifurcation in terms of the design variables x,y and the bifurcation parameter *S*. Here, the bifurcation of interest is the saddle-node bifurcation (also denoted as fold or limit point bifurcation). In particular, there are 4 saddle-node bifurcations in the mushroom-shaped diagram (as indicated in figure below).

A parametric condition for saddle-nodes in biochemical networks with mass action kinetics was demonstrated by.^40^^,^^41^ This analytic condition is exploited in effective computational tools to detect, via optimization, saddle-node bifurcations and bistability in biochemical reaction networks with mass action kinetics.^9^^,^^10^^,^^11^ For non-mass-action kinetics, as it is the case in this study, we use an alternative formulation of optimization problem (see^42^), in which the objective function to minimize is defined in terms of the extended Jacobian of the system, as follows.

Starting from Equation 2 we compute the following extended Jacobian:

(Equation 3) $$Q=\left[D_{\omega }f\left(\omega ,x,y,S\right),D_{S}f\left(\omega ,x,y,S\right)\right]$$

with dimensions N×N+1. This extended Jacobian can be obtained symbolically or approximated numerically by finite differences. The tangent vector at the bifurcation point can be computed as β=null(Q). By definition, a saddle-node point occurs when $\beta_{N+1}=0$, i.e., if the N+1 entry of the tangent vector β is equal to zero (see figure in bifurcation conditions). Therefore, the function:

(Equation 4) $$J=\beta_{N+1}^{2}\left(\dot{\omega },\omega ,y,S\right)$$

reaches its minimum value (i.e. J = 0) at a saddle node point.

#### Optimization-based automated design

Mushroom-shaped bifurcation diagrams show four saddle-node bifurcation points, see figure in bifurcation conditions. in which SN1, SN2, SN3 and SN4 indicate saddle node bifurcations with coordinates ($S_{i},\omega_{i}$) for i=1,…,4 in the bifurcation diagram. Following the reasoning of the previous subsection, the function:

$$\Psi =\sum_{k=1}^{4}J_{k}\;\text{with}\;J_{k}=\beta_{kN+1}^{2}\left({\dot{\omega }}_{k},\omega_{k},x,y,S_{k}\right)$$

reaches its minimum (Ψ=0) when four saddle-node bifurcations occur at coordinates ($S_{k},\omega_{k}$) for k=1,…,4 in the bifurcation diagram. Therefore, we can formulate the design of bio-circuits with mushroom bifurcation behavior (with saddle node bifurcations located within the desired regions in the bifurcation diagram) as the following optimization problem:

$$\min_{x,y,S}\;\Psi \left(\dot{\omega },\omega ,x,y,S\right)$$

subject to:

(Equation 5) f(ω,x,y,S)=0

(Equation 6) $$\omega_{L}\leq \omega \leq \omega_{U}$$

(Equation 7) $$\begin{array}{l} x_{L}\leq x\leq x_{U}\\ y_{L}\leq y\leq y_{U}\\ S_{L}\leq S\leq S_{U} \end{array}$$

Note that *S* here denotes a four dimensional vector, being $S_{L}$ and $S_{U}$ the lower and upper bounds of the target rectangular regions. The lower and upper bounds of the real and integer decision variables are encoded in vectors $x_{L},y_{L}$ and $x_{U},y_{U}$, respectively. We can specify the (rectangular) regions in the bifurcation diagram where we want the bifurcation points to be located through the values of the lower and upper bounds of the inequality constraints (6) and the decision variable S, see for example the rectangular regions in Figure 1C. The choice of constraints can be defined according with the design specifications required by the user, related to the desired specific functionality (for example, if the desired mushroom might be inverted, i.e. upside down), the particular experimental constraints, etc. In this work, the goal was to find circuits leading to mushrooms (including the possibility of inverted ones), so we defined 2 instead of 4 rectangular regions as constraints, which led to a very efficient search for biocircuits with the target behavior). The constraints used are in Tables S1 and S2. Note that the constraints for u and v are different, resulting in a break of the symmetry of the model.

The optimization problem is a Mixed Integer Nonlinear Programming problem (MINLP). Due to its non-convex nature, global optimization solvers are needed to obtain adequate solutions.^43^ It is important to remark that the solution of this optimization problem is not unique, since there are multiple combinations of topology and parameters leading to mushroom bifurcations. A single run of the algorithm will find a particular solution very efficiently (in the order of a few seconds for a standard PC), whereas a multistart strategy (running the optimization algorithm multiple times) will effectively find different topologies with mushroom bifurcation diagram.

For the screening of 2-gene and 3-gene topologies with mushroom behavior, we use a multistart strategy with 2000 and 10000 runs, respectively. As indicated in the results section, the corresponding bounds for the parameters for the 2-gene and 3-gene networks are included in the Tables S1–S3, and the solution sets denoted by *Mushroom2D* and *Mushroom3D*, respectively.

In advanced design applications, it is usually the case that multiple objectives need to be taken into account.^6^ For example, during the design of next generation biosensors, we might be interested not only in a mushroom-shaped diagram, but also in maximizing the regions of bistability. In numerous occasions, it is also required to operate at low protein burden to avoid compromising the viability of the cells, being the minimal protein production cost another important objective to be considered. In order to find the best designs with respect to multiple criteria ($\psi_{1},\ldots ,\psi_{M}$), we consider a multi-objective formulation of the optimization problem., i.e. we set the mushroom bifurcation condition Ψ=0 as a constraint of the following Multiobjective Mixed Integer Nonlinear Programming (MO-MINLP) problem:

$$\min_{x,y,S}\;\psi_{1}\left(\dot{\omega },\omega ,x,y,S\right),\ldots ,\psi_{M}\left(\dot{\omega },\omega ,x,y,S\right)$$

subject to:

f(ω,x,y,S)=0

$$\Psi \left(\dot{\omega },\omega ,x,y,S\right)=0$$

(Equation 8) $$\begin{array}{l} x_{L}\leq x\leq x_{U}\\ y_{L}\leq y\leq y_{U}\\ S_{L}\leq S\leq S_{U} \end{array}$$

The solution of the above multicriteria mixed integer nonlinear problem is not unique, but a set of vectors representing the best compromises between the (usually conflicting) objective functions. This set of best trade-offs is usually known as the Pareto set.

The isola-behavior can be also designed automatically, taking into account that at the isola both bifurcation curves of steady-state solutions $u^{*}\left(S\right)$ exhibit two saddle-node bifurcations at the coordinates $\left(S_{1},u_{1}^{*}\right)$ and $\left(S_{2},u_{2}^{*}\right)$ with $S_{1}<S_{2}$. The isola will fulfill the property $u_{SS}^{-1}\left(S_{1}\right)>0$ and $u_{SS}^{-1}\left(S_{2}\right)<0$, where $u_{SS}^{-1}$ is the local curvature of the inverse of the function $u^{*}\left(S\right)$ that can be evaluated numerically. In contrast, for a bistable switch we would expect $u_{SS}^{-1}\left(S_{1}\right)<0$ and $u_{SS}^{-1}\left(S_{2}\right)>0$. These conditions are easily incorporated into the optimization problem in order to target the isola bifurcation.

#### Optimization strategy and solvers

Computing the Pareto set of optimal designs by solving the above problems efficiently and reliably can be a daunting task due to their non-convexity, arising from their highly constrained, partially discrete and non-linear nature.

Here we transform the multicriteria mixed integer formulation (MO-MINLP) into a finite set of single-objective mixed integer (MINLP) problems by adopting an ε-constraint approach.^44^ The resulting set of MINLPs is then solved using a hybrid strategy, eSS-MISQP, which combines a diversification phase (using a global optimization metaheuristic, eSS) with intensification steps (using an efficient local mixed integer optimization solver, MISQP). This hybrid strategy has been found to outperform other global approaches in terms of both efficiency and robustness.^6^

### Quantification and statistical analysis

#### Analysis of robustness

In order to assess the robustness of a topology, we used two different scores based on the covariance and the interquantile range of the successful parameter sets for that topology (see Figure 3). On the one hand we defined a robustness scores based on the hypervolume of the parameter space occupied by solutions that return a successful mushroom bifurcation. This is achieved by calculating the logarithm of the determinant of the covariance matrix of the parameter sets. On the other hand we used a score successfully employed for gene regulatory structures^45^ calculated as the sum of the interquantile ranges (IQR) measuring the spread of the distributions of each of the parameters for the solution set. Both scores were regularized by using the standardized parameters of the aggregated parameter set for all the topologies. The robustness histograms for the extremes of the Pareto front in Figure 3 are included in the Figure S4.

## Data Availability

• Data files including the resulting topologies and parameters from the optimization are available online at Zenodo: https://doi.org/10.5281/zenodo.6024249. • The code used in the analysis can be found in the same repository at Zenodo: https://doi.org/10.5281/zenodo.6024249. • Any additional information required to reanalyse the data reported in this work is available from the lead contact upon reasonable request.
