# Supplementary material for: Automated design of gene circuits with optimal mushroom-bifurcation behavior
Source: iScience. 2023 May 9;26(6):106836. doi: 10.1016/j.isci.2023.106836 (PMC10225937; doi:10.1016/j.isci.2023.106836)
Supplement: Document S1. Figures S1–S4 and Tables S1–S6 [file mmc1.pdf]

iScience, Volume *26*

Supplemental information

Automated design of gene circuits with optimal mushroom-bifurcation behavior

Irene Otero-Muras, Ruben Perez-Carrasco, Julio R. Banga, and Chris P. Barnes

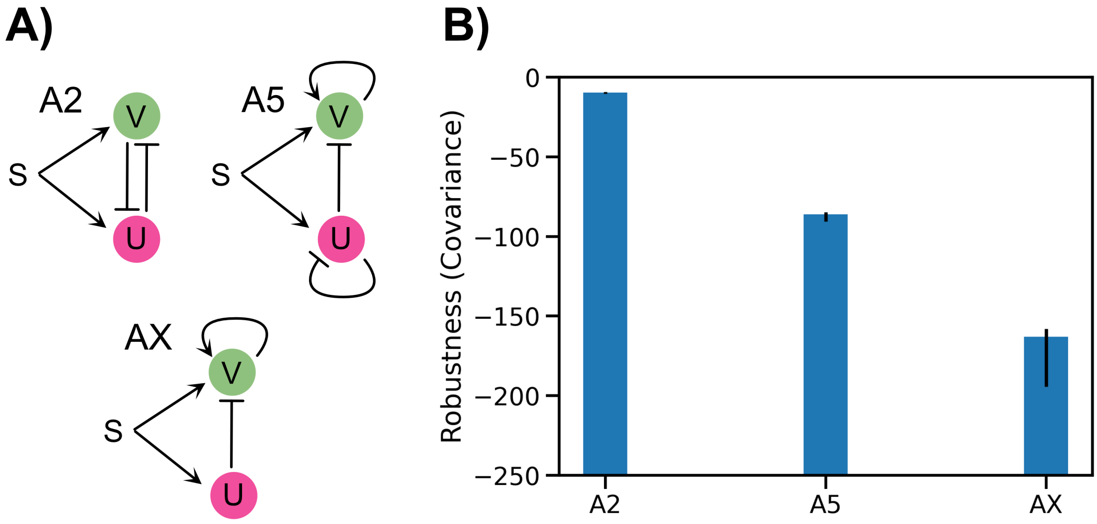

Figure S1: Comparison of robustness of two of the topologies found in the study with the topology analyzed in (Dey & Barik 2021). A) The three topologies compared in the analysis. Topology AX corresponds with the topology studied in (Dey & Barik 2021). B) Robustness analysis of the 3 topologies. Related to Fig. 2.

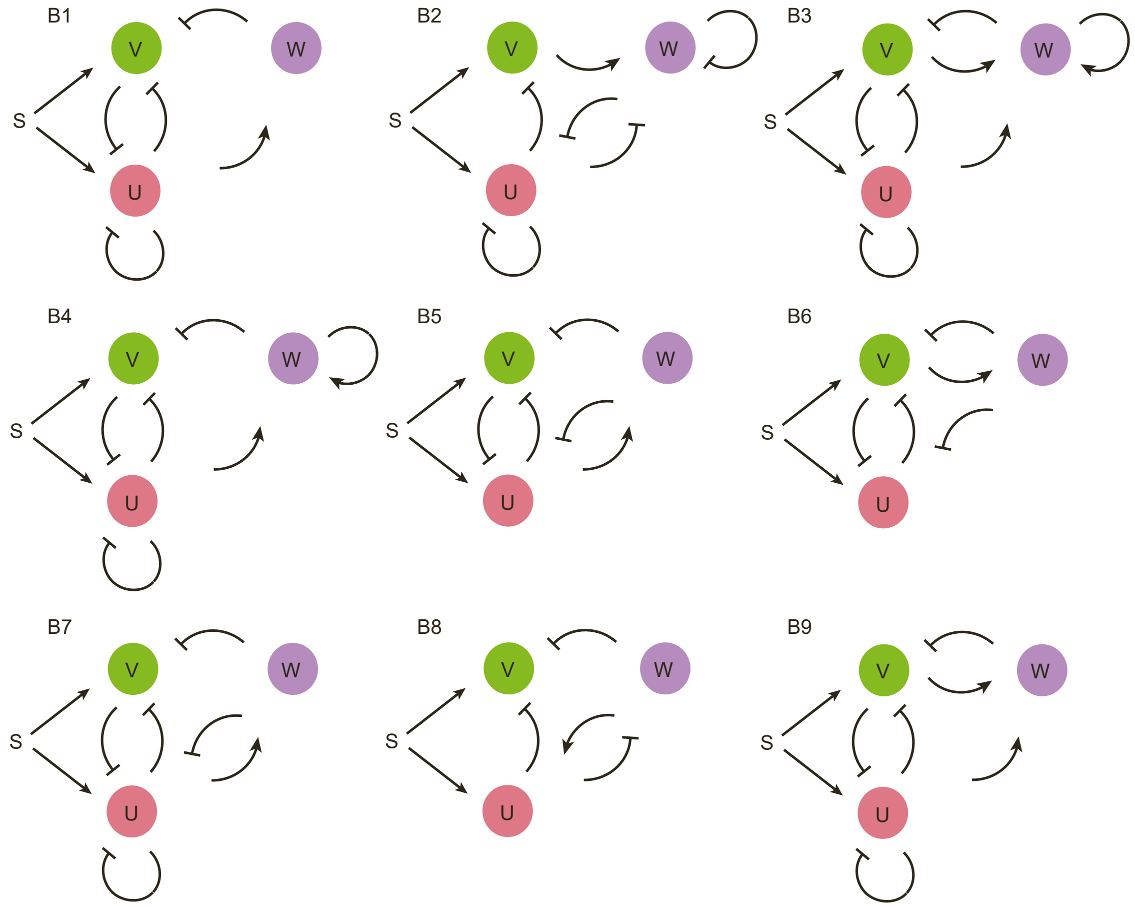

Figure S2: Most frequent 3 gene topologies (Related to Figure 3 and Table S3).

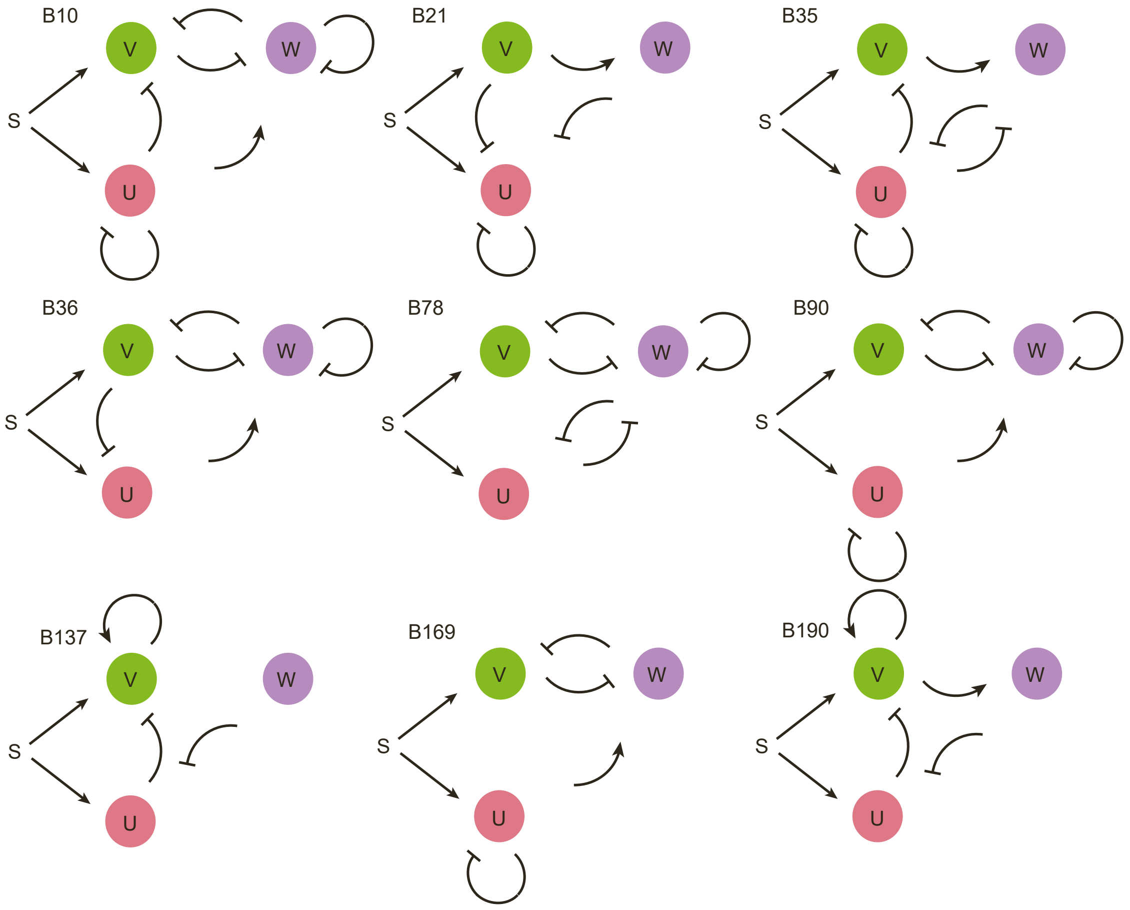

Figure S3: Selection of 3-gene topologies (Related to Figure 3 and Table S4).

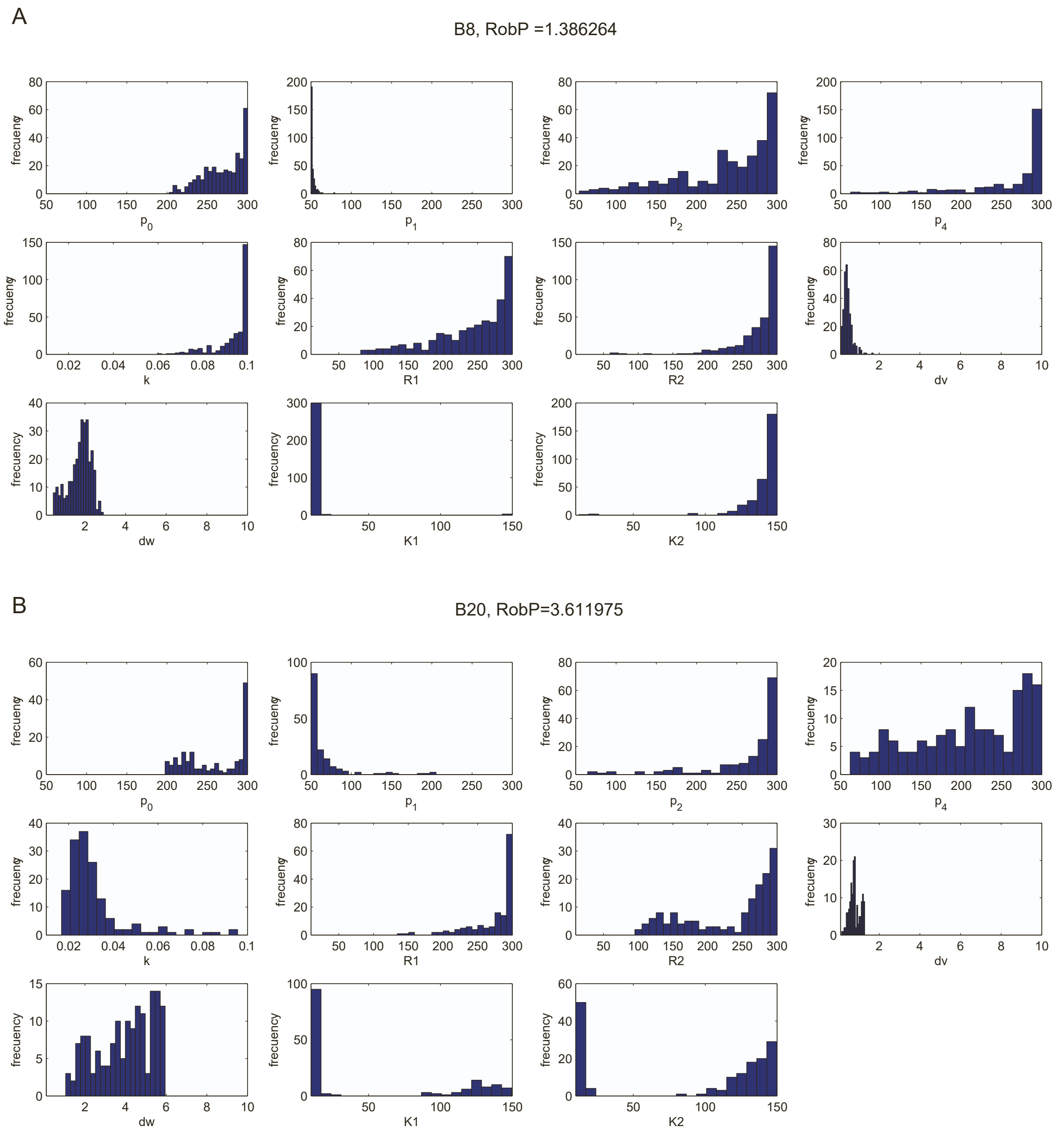

Figure S4: Robustness proxy histograms for the two extremes of the Pareto Front in the main text Fig. 3 B. The minimum and maximum x-axis values are the lower and upper bounds for the parameters, respectively. Related to Fig. 3.

Table S1: Upper and Lower bounds (UB and LB respectively) for the decision variables of the optimization problem, including the parameters and the states at the bifurcation points $S1$ and $S2$ (2 dimensional gene regulatory network). Related to Fig. 2.

	p_0	p_1	p_2	k	R_1	R_2	dv	$K1$	$K2$	S_1	S_2
UB	300	300	300	0.1	300	300	10	150	150	50	200
LB	50	50	50	0.01	10	10	0.1	10	10	5	80

	u (S_1)	v (S_1)	u (S_2)	v (S_2)
UB	100	200	100	200
LB	50	10	50	10

Table S2: Upper and Lower bounds (UB and LB respectively) for the decision variables of the optimization problem, including the parameters and the states at the bifurcation points $S1$ and $S2$ (3 dimensional gene regulatory network). Related to Fig. 3.

	p_0	p_1	p_2	p_4	k	R_1	R_2	dv	dw	$K1$	$K2$	S_1	S_2
UB	300	300	300	300	0.1	300	300	10	10	150	150	50	200
LB	50	50	50	50	0.01	10	10	0.1	0.1	10	10	5	80

	u (S_1)	v (S_1)	w (S_1)	u (S_2)	v (S_2)	w (S_2)
UB	100	200	100	100	200	100
LB	50	10	50	50	10	50

Table S3: Most frequent 2-gene topologies obtained with the robustness analysis. The corresponding graphs can be found in Fig. 2. The covariance robustness score (the closer to zero the more robust) is also indicated for the structures with more than 100 hits. Related to Fig. 2.

Structure	Signature	Frequency	Robustness (covariance)
A1	---0	1430	-16.9
A2	0--0	205	-8.98
A3	0---	130	-12.6
A4	---+	117	-18.67
A5	-0-+	15	N/A
A6	+--0	3	N/A
A7	+-0-	1	N/A

Table S4: Most frequent 3-gene topologies obtained with the robustness analysis. The corresponding graphs can be found in Fig. S2. Related to Fig. 3.

Structure	Signature	Frequency
B1	- - 0 - 0 - + 0 0	812
B2	- 0 - - 0 0 - + -	499
B3	- - 0 - 0 - + + +	483
B4	- - 0 - 0 - + 0 +	352
B5	0 - - - 0 - + 0 0	326
B6	0 - - - 0 - 0 + 0	312
B7	- - - - 0 - + 0 0	306
B8	0 0 - - 0 0 0 + -	304
B9	- - 0 - 0 - + + 0	260

Table S5: Topologies of 3-gene regulatory networks showing mushroom bifurcation behaviour (and that, additionally, are not built up from 2-gene mushroom structures). Graphs for the first 9 topologies are depicted in Fig. S2. Related to Fig. 3.

Structure	Signature	Frequency	Structure	Signature	Frequency
B2	- 0 - - 0 0 - + -	499	B90	- 0 0 0 0 - + - -	12
B8	0 0 - - 0 0 0 + -	304	B95	0 0 - - + 0 + + 0	11
B10	- 0 0 - 0 - + - -	258	B96	0 0 - - 0 0 - + -	11
B19	0 - - - 0 - + + -	152	B97	0 - 0 0 + - + - -	11
B21	- - - 0 0 0 0 + 0	143	B102	- - - 0 + - + - -	11
B22	- - - 0 0 0 + + 0	133	B115	0 - 0 0 - - + - -	8
B35	- 0 - - 0 0 - + 0	69	B137	0 0 - - + 0 0 0 0	6
B36	0 - 0 0 0 - + 0 -	66	B150	0 0 - - + 0 0 + 0	5
B38	- 0 - - 0 - + - -	57	B155	- 0 - - + 0 0 + +	5
B49	- 0 0 - 0 - + - 0	29	B157	- - - 0 0 - + - -	5
B68	- 0 - 0 0 - + - 0	17	B160	+ 0 - - 0 0 + + -	4
B76	- 0 - - + 0 + + 0	15	B161	0 - 0 0 - - + - 0	4
B78	0 0 - 0 0 - + - -	14	B169	- 0 0 0 0 - + - 0	4

Table S6: Proxy of Robustness for the first 5 structures in Table S4 (the corresponding topologies are depicted in Fig. S3). Related to Fig. 3.

Structure	Signature	dd
B2	- 0 - - 0 0 - + -	0.9331
B8	0 0 - - 0 0 0 + -	1.3863
B10	- 0 0 - 0 - + - -	3.0162
B19	0 - - 0 0 - + + -	1.8540
B21	- - - 0 0 0 0 + 0	1.6140
B22	- - - 0 0 0 + + 0	1.6726
